# Data-Sharing Statements Requested from Clinical Trials by Public, Environmental, and Occupational Health Journals: Cross-Sectional Study

**DOI:** 10.2196/64069

**Published:** 2025-02-07

**Authors:** Yingxin Liu, Jingyi Zhang, Lehana Thabane, Xuerui Bai, Lili Kang, Gregory Y H Lip, Harriette G C Van Spall, Min Xia, Guowei Li

**Affiliations:** 1 Center for Clinical Epidemiology and Methodology The Affiliated Guangdong Second Provincial General Hospital of Jinan University Guangzhou China; 2 Department of Health Research Methods, Evidence, and Impact McMaster University Hamilton, ON Canada; 3 Father Sean O'Sullivan Research Centre St Joseph's Healthcare Hamilton Hamilton, ON Canada; 4 Faculty of Health Sciences University of Johannesburg Johannesburg South Africa; 5 Department of Epidemiology School of Medicine Jinan University Guangzhou China; 6 School of Public Health Guangdong Pharmaceutical University Guangzhou China; 7 Liverpool Centre for Cardiovascular Sciences at University of Liverpool Liverpool Heart & Chest Hospital Liverpool John Moores University Liverpool United Kingdom; 8 Department of Clinical Medicine Aalborg University Aalborg Denmark; 9 Department of Medicine McMaster University Hamilton, ON Canada; 10 Guangdong Provincial Key Laboratory of Food, Nutrition and Health & Department of Nutrition School of Public Health Sun Yat-sen University Guangzhou China

**Keywords:** data sharing, clinical trial, public health, International Committee of Medical Journal Editors, ICMJE, journal request, clinical trials, decision-making, occupational health, health informatics, patient data

## Abstract

**Background:**

Data sharing plays a crucial role in health informatics, contributing to improving health information systems, enhancing operational efficiency, informing policy and decision-making, and advancing public health surveillance including disease tracking. Sharing individual participant data in public, environmental, and occupational health trials can help improve public trust and support by enhancing transparent reporting and reproducibility of research findings. The International Committee of Medical Journal Editors (ICMJE) requires all papers to include a data-sharing statement. However, it is unclear whether journals in the field of public, environmental, and occupational health adhere to this requirement.

**Objective:**

This study aims to investigate whether public, environmental, and occupational health journals requested data-sharing statements from clinical trials submitted for publication.

**Methods:**

In this bibliometric survey of “Public, Environmental, and Occupational Health” journals, defined by the Journal Citation Reports (as of June 2023), we included 202 journals with clinical trial reports published between 2019 and 2022. The primary outcome was a journal request for a data-sharing statement, as identified in the paper submission instructions. Multivariable logistic regression analysis was conducted to evaluate the relationship between journal characteristics and journal requests for data-sharing statements, with results presented as odds ratios (ORs) and corresponding 95% CIs. We also investigated whether the journals included a data-sharing statement in their published trial reports.

**Results:**

Among the 202 public, environmental, and occupational health journals included, there were 68 (33.7%) journals that did not request data-sharing statements. Factors significantly associated with journal requests for data-sharing statements included open access status (OR 0.43, 95% CI 0.19-0.97), high journal impact factor (OR 2.31, 95% CI 1.15-4.78), endorsement of Consolidated Standards of Reporting Trials (OR 2.43, 95% CI 1.25-4.79), and publication in the United Kingdom (OR 7.18, 95% CI 2.61-23.4). Among the 134 journals requesting data-sharing statements, 26.9% (36/134) did not have statements in their published trial reports.

**Conclusions:**

Over one-third of the public, environmental, and occupational health journals did not request data-sharing statements in clinical trial reports. Among those journals that requested data-sharing statements in their submission guidance pages, more than one quarter published trial reports with no data-sharing statements. These results revealed an inadequate practice of requesting data-sharing statements by public, environmental, and occupational health journals, requiring more effort at the journal level to implement ICJME recommendations on data-sharing statements.

## Introduction

Data sharing plays a crucial role in health informatics, contributing to improving health information systems, enhancing operational efficiency, informing policy and decision-making, and advancing public health surveillance including disease tracking [1]. Specifically, sharing individual patient data (IPD) from clinical trials promotes scientific advancement, enhances transparent reporting, and meets ethical obligations to trial participants [1,2]. In 2017, the International Committee of Medical Journal Editors (ICMJE) required that all papers submitted after July 1, 2018, must include a data-sharing statement [3].

Data sharing is of special importance for trials in the field of public, environmental, and occupational health, where findings can translate to policies with population-level impact. Given the fact that many trials incorporate public engagement and are commonly designed as community-based cluster trials, IPD sharing may improve public trust and support by enhancing transparent reporting and reproducibility and helping patients access their health data information and make informed health care decisions [4]. While public perception and willingness to share IPD are generally affected by concerns about privacy, transparency is a critical step toward getting buy-in from the public, particularly since environmental and public health policies may have implications on individual rights [4-6]. Specifically, addressing the ethical and regulatory issues surrounding the confidentiality of shared data is crucial for actual data sharing [7]. Nonetheless, enhancing transparency and providing detailed information about how data will be collected, used, and shared can effectively improve trust among individuals and facilitate actual data sharing. In the setting of public health emergencies such as epidemics, timely data sharing fosters societal-level preparedness, helps advance discovery in disease prevention and therapy, and allays fear of the unknown [8]. Of note, challenges and barriers to data sharing remained for public health emergencies, including poor accessibility, language difficulties, mistrust toward the health system, or concerns about insurance eligibility [4]. Global policymakers and researchers have advocated data sharing for the COVID-19 pandemic and developed publicly available platforms to enhance the sharing of IPD [9-11]. Nevertheless, a study based on trial registration information showed that the proportion of intent to share IPD for COVID-19 clinical trials did not differ from the contemporary non–COVID-19 trials [12].

Some previous studies explored journal requests for data-sharing statements in high-profile journals or specific clinical fields, with no focus on the field of public, environmental, and occupational health [13-16]. It is unclear whether public, environmental, and occupational health journals adhere to ICMJE’s requirement for data-sharing statements. Clinical trials are the gold standard for causal inference, and their data sharing is particularly relevant as trials change policy and practice. Therefore, we conducted a survey to investigate whether public, environmental, and occupational health journals request data-sharing statements in clinical trial submissions. We also explored associations between journal characteristics and journal requests for data-sharing statements.

## Methods

### Study Design

We followed the Strengthening the Reporting of Observational Studies in Epidemiology (STROBE) guideline to report this study [17]. According to previous publications exploring the field of public, environmental, and occupational health, we included all 400 journals that were in the category of “Public, Environmental, and Occupational Health” defined by the Journal Citation Reports (as of June 2023) [18-20]. Subsequently, we searched the journal webpages using the keywords “trial” or “clinical trial” to assess whether the journal had published clinical trial reports between January 1, 2019, and December 31, 2022. Journals that did not publish clinical trial reports with IPD between 2019 and 2022 were excluded because the ICMJE required data-sharing statements in clinical trials after July 2018. A total of 202 journals were included for analysis (Figure S1 in Multimedia Appendix 1).

### Study Outcome

The study outcome was a journal request for data-sharing statements. We searched journals’ webpages about their data-sharing statement request by using the keywords “data sharing,’’ ‘‘data availability,’’ “research data,” “data accessibility,” “data deposition,” and ‘‘data deposit.” Google Translate was used for journals that were in non-English and non-Chinese languages. A journal was deemed to request data-sharing statements if the journal clearly asked trial authors to provide a data-sharing statement on the paper submission instructions. Table S1 in Multimedia Appendix 1 shows the list of descriptions of data-sharing statement requests from journals’ paper submission instructions.

Although some journals did not request statements on the paper submission instructions, it was possible that the journals would request trial authors to upload a statement or ask the authors about their willingness to share data in the journal submission system. These journals were also considered to have requests for data-sharing statements. A random sample of 10 journals that had no request on their submission guidance pages was used for the mockup submission process. Specifically, we evaluated whether these journals without requests from their submission guidance would ask for information on data-sharing statements from trial authors in their submission systems. None of these journals were found to request a data-sharing or availability statement in their submission systems.

The journals with data-sharing statement requests differed in how strongly they worded the request in the description. Specifically, if a journal used the terms “encourage” or “recommend” (eg, we encourage the use of data-sharing statements), we categorized the type of journal request for data-sharing statements as Weak. If a journal worded the terms “require,” “request,” “should,” “mandate,” or “must” (eg, all research articles must contain a data-sharing statement), we categorized the journal request as Strong (Table S1 in Multimedia Appendix 1). These terms used to classify the type of journal requests for data-sharing statements were mainly adopted from previous literature [16]. To minimize the potential for misclassification, we performed data extraction and categorized the type of journal request in duplicate (YL and JZ). Any disagreement was resolved by discussion or in consultation with a senior investigator (GL).

### Data Extraction

Data extraction was performed by study investigators in duplicate and pairs (YL and JZ, XB, and LK) from September 1, 2023, to March 31, 2024. If there was a disagreement between these study investigators, we tried to reach a consensus by double-checking the extracted information and discussing it with the group. If a consensus could not be reached, we consulted with a senior investigator for a decision (GL). Data on journal characteristics were extracted, including the percentage of open access, its publication region, publisher, publication language, journal impact factor in 2022 (released in June 2023), Journal Citation Reports quartile, whether the journal was on the ICMJE list, whether the journal explicitly endorsed the Consolidated Standards of Reporting Trials (CONSORT) in its submission guidance page, and the total number of trials published between 2019 and 2022 for each journal.

The percentage of open access, as extracted from the open access section in the Journal Citation Reports, denoted the percentage of open access items among all the citable items published in the journal in the previous 3 years.

Both data on publishers and publication regions were collected from the Journal Citation Reports. Journals from the same publisher could belong to different publication regions, thereby potentially providing more information on social and cultural differences. For example, 3 journals (*Disability and Health Journal*, *Cancer Epidemiology*, and *International Journal of Hygiene and Environmental Health*) belonged to Elsevier, which has its headquarters in Amsterdam (Netherlands), while these journals were published in the United States, United Kingdom, and Germany, respectively. Therefore, we collected both the data on the publisher and the publication region of each journal for potential inclusion in analyses.

### Statistical Analysis

Continuous characteristics were described using medians with lower and upper quartiles (Q1-Q3), and categorical variables using counts and percentages. Comparisons between journals with and without data-sharing statement requests were conducted by *t* test and chi-square test for continuous and categorical variables, respectively.

**Figure 1:**
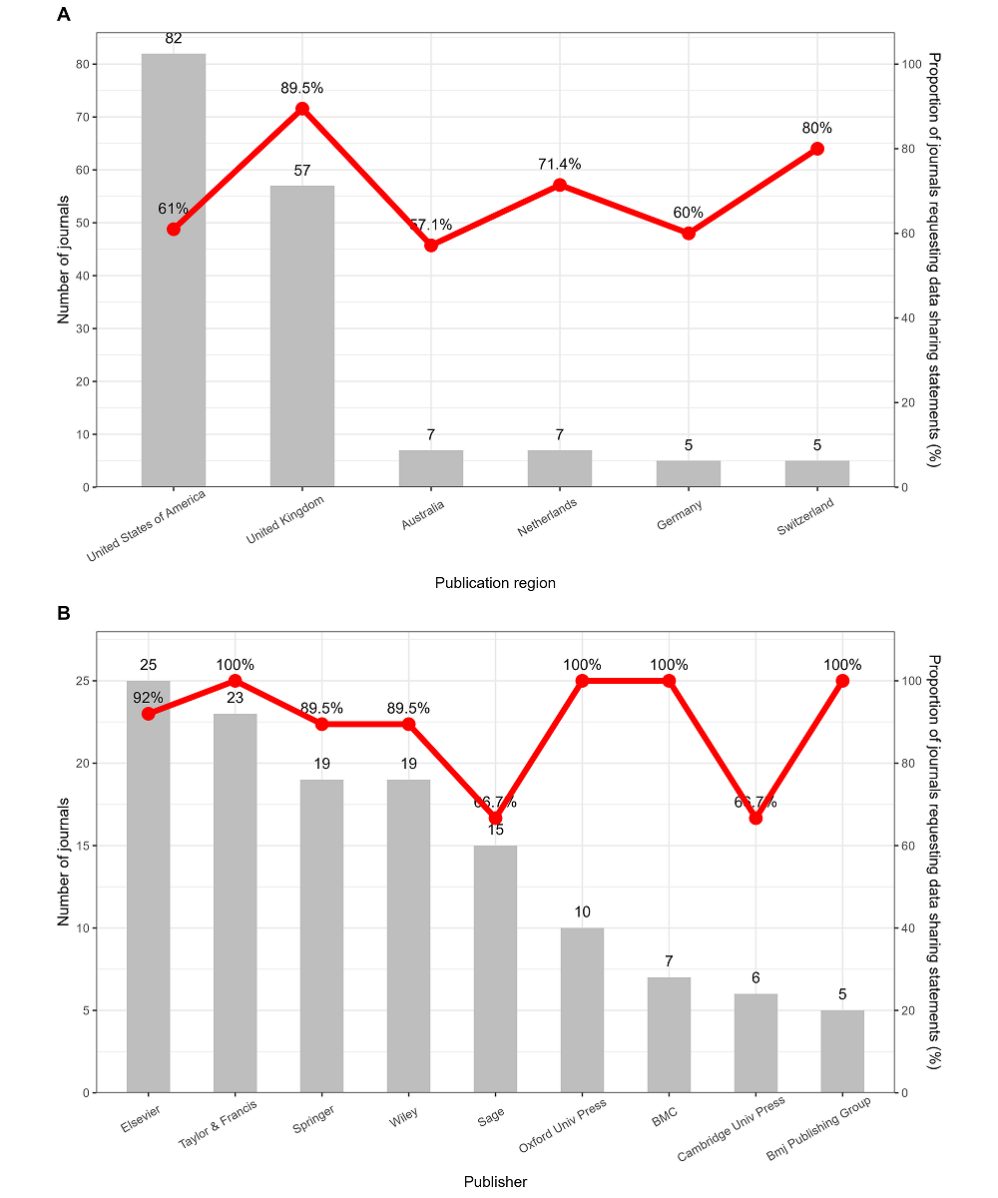
Characteristics of journals according to data sharing statement request by publication region and publisher. Only publication regions and publishers with ≥5 journals were listed. Proportions were presented as lines. (A) The count and proportion of journals according to data sharing statement request by publication region. (B) The count and proportion of journals according to data sharing statement request by publisher.

Multivariable logistic regression analysis was conducted to evaluate the relationship between journal characteristics and a journal’s request for data-sharing statements. Some journal characteristics were considered as covariates in the model including publication region (United Kingdom and United States vs others), publisher, and Journal Citation Reports quartile (Q1-Q2 vs Q3-Q4), in which these characteristics had been reported to associate with journal request in previous studies [15,16]. In this survey, the United States and the United Kingdom had the top 2 numbers of public, environmental, and occupational health journals, far more than the remaining publication regions (Figure 1A). Therefore, we treated the variable of publication region as 3 levels (United States, United Kingdom, and others) and took the other regions as the reference group. Other characteristics were also adjusted for in the model based on our expertise and group discussion, including open access (yes vs no, taking 50% of open access as threshold), journal impact factor (≥ vs <2.9, taking the median of 2.9 as a threshold), whether the journal was on the ICMJE list (yes vs no), whether the journal endorsed the CONSORT (yes vs no), and the number of trials published between 2019 and 2022 (≥ vs <11, taking the median of 11 as a threshold). We assessed the potential multicollinearity in the model by calculating variance inflation factor (VIF) values for each journal characteristic, with a VIF >4 indicating severe multicollinearity [21]. Two variables, the Journal Citation Reports quartile and publisher, were removed because they had a VIF >4 (6.8 for Journal Citation Reports quartile and 4.3 for publisher), leaving all the other journal characteristics remaining in the final model. Results were presented as odds ratios (ORs) with corresponding 95% CIs. An OR >1 indicated that the variable was associated with increased odds of having requests for data-sharing statements.

Two sensitivity analyses were conducted to evaluate the robustness of the main findings. First, journals from the same publisher may share a similar or same journal request for data-sharing statements. Given the removal of the publisher from our main analysis due to its VIF, to account for this cluster effect, we performed a sensitivity analysis by using the generalized estimating equations (GEE) approach with an exchangeable correlation structure to model the intraclass correlation of the journals within the same publisher [22]. GEE is specifically designed to handle correlated data from clustered or repeated measures. By using an exchangeable correlation structure, GEE allows for the assumption that the correlation between any 2 observations within the same cluster (eg, publisher) is constant. This could accurately reflect the underlying data structure and improve the validity of the statistical inferences made [22]. Furthermore, we performed another sensitivity analysis by using the continuous forms for three journal characteristics (percentage of open access, journal impact factor, and number of trials published between 2019 and 2022) for the multivariable regression analysis. Using continuous forms in the model could make full use of the information provided by the continuous variables, which may therefore generate estimates with larger uncertainty when compared with the model using categorical forms [23]. Thus this approach could test the robustness of our main findings from multivariable regression analysis.

We further performed 3 exploratory analyses for this survey. First, we evaluated the relationship between journal characteristics and the type of journal request for data-sharing statements (weak or strong). Multinomial logistic regression analysis was used for this analysis, taking no request as the reference.

Second, to assess whether the journal request for data-sharing statements from the paper submission instructions was in line with their published clinical trial reports, we extracted information on data-sharing statements from 3 recent clinical trial reports published after September 1, 2023. We identified the recent trial reports by searching PubMed in descending order by publication date for each journal. We determined whether there was any data-sharing statement in these clinical trial reports by thoroughly searching the reports, the web pages where the journals published these reports, and their supplemental materials. Journals were therefore categorized into having any or no data-sharing statements in their published trial reports. We used the McNemar test to explore whether there was a significant discordance between journal requests (identified on the paper submission instructions) and the publication of a data-sharing statement (in their published trial reports). Furthermore, multivariable logistic regression was used to evaluate journal characteristics in relation to journals having data-sharing statements in trial reports, taking journals having none of the statements as a reference.

We conducted a third exploratory analysis trying to explore the temporal trend of journal requests for data-sharing statements. The study by Siebert et al [15] included the largest number of journals from the “Clinical Medicine” group defined by Journal Citation Reports in 2018 (n=489), among previous studies assessing journal requests for data-sharing statements. We matched our included journals with this previous publication to evaluate any potential difference in journal requests for data-sharing statements from 2018 to 2023 by using the McNemar mid-*P* test [24]. A significant difference would indicate there was a significant increase or decrease in journal requests for data-sharing statements over the past 5 years.

All statistical tests were 2-sided with a significance level of .05. Analyses were conducted in R software (version 4.1.0; R Core Development Team) and SAS software (version 9.4; SAS Institute).

### Ethical Considerations

The present analysis was a secondary analysis based on published materials and website. In accordance with current regulations and the hospital's policy, studies that do not involve human participants or animals are exempt from the requirement for formal ethical approval.

## Results

We included a total of 202 public, environmental, and occupational health journals for analysis (Figure S1 in Multimedia Appendix 1). As shown in Table 1, the included journals were mainly non–open access (71.8%) and published in the English language (96.0%). Overall, 18.8% of the journals were on the ICMJE list and 60.4% endorsed CONSORT. The United States (40.6%) and the United Kingdom (27.7%) had the top 2 numbers of journals among all publication regions, while Elsevier, Taylor & Francis, Springer, Wiley, and Sage were the top 5 publishers with the largest number of journals (Figure 1 and Figures S2 and S3 in Multimedia Appendix 1). The median journal impact factor was 2.9 (Q1-Q3: 1.9-4.5), and the median number of trials published between 2019 and 2022 was 11.0 (5.0-26.8).

**Table 1:** Journals’ characteristics and comparisons according to data-sharing statement request. Results are shown as count (%) unless otherwise specified.

Journal characteristics		Overall (n=202)	Whether journal requested data-sharing statements		*P* value
			No (n=68)	Yes (n=134)	
**Publication language**					
	English, n (%)	194 (96)	60 (88.2)	134 (100)	<.001
	Non-English, n (%)	8 (4)	8 (11.8)	0 (0)	.98
	Percentage of open access (%), median (Q1-Q3)	21.4 (9.4-76.7)	14.0 (0.2-90.4)	23.3 (11.7-48.2)	
**Open access^b^, n (%)**					
	No	145 (71.8)	44 (64.7)	101 (75.4)	.11
	Yes	57 (28.2)	24 (35.3)	33 (24.6)	
**Publication region, n (%)**					
	United States	82 (40.6)	32 (47.1)	50 (37.3)	<.001
	United Kingdom	56 (27.7)	5 (7.4)	51 (38.1)	
	Others	64 (31.7)	31 (45.6)	33 (24.6)	
**Publisher, n (%)**					
	Elsevier	25 (12.4)	2 (2.9)	23 (17.2)	<.001
	Taylor & Francis	23 (11.4)	0	23 (17.2)	
	Springer	19 (9.4)	2 (2.9)	17 (12.7)	
	Wiley	19 (9.4)	2 (2.9)	17 (12.7)	
	Sage	15 (7.4)	5 (7.4)	10 (7.5)	
	Others	101 (50)	57 (83.8)	44 (32.8)	
	Journal impact factor, median (Q1-Q3)	2.9 (1.9-4.5)	2.1 (1.3-3.5)	3.3 (2.3-4.8)	.16
	Journal impact factor ≥2.9^b^	99 (49.0)	24 (35.3)	75 (56.0)	.01
**Journal Citation Reports quartile**					
	Q1-Q2, n (%)	92 (45.5)	23 (33.8)	69 (51.5)	.02
	Q3-Q4, n (%)	110 (54.5)	45 (66.2)	65 (48.5)	
	Number of trials published between 2019 and 2022, median (Q1-Q3)	11.0 (5.0-26.8)	8.00 (4.0-15.0)	14.0 (6.0-30.0)	.01
	Number of trials published ≥11^c^, n (%)	103 (51)	29 (42.6)	74 (55.2)	.09
**Whether the journal was on the ICMJE^d^ list, n (%)**					
	No	164 (81.2)	55 (80.9)	109 (81.3)	.94
	Yes	38 (18.8)	13 (19.1)	25 (18.7)	
**Whether the journal endorsed CONSORT^e^, n (%)**					
	No	80 (39.6)	38 (55.9)	42 (31.3)	<.001
	Yes	122 (60.4)	30 (44.1)	92 (68.7)	

^a^Not applicable.

^b^Open access journal was defined as having a percentage of open access ≥50%.

^c^The median journal impact factor was 2.9; the median number of trials published between 2019 and 2022 was 11.

^d^ICMJE: International Committee of Medical Journal Editors.

^e^CONSORT: Consolidated Standards of Reporting Trials.

There were 134 (66.3%) journals requesting data-sharing statements in clinical trials (Table 1). Journals requesting data-sharing statements had a higher journal impact factor and a larger proportion of being in the Journal Citation Report Q1-Q2. They were more likely to endorse CONSORT and publish more clinical trials compared with journals that did not request data-sharing statements. Significant differences were also found in publication regions and publishers between journals with and without request. As displayed in Figure 1A, the United Kingdom (89.5%) had the largest proportion of journals requesting data-sharing statements among all the publication regions.

**Figure 2:**
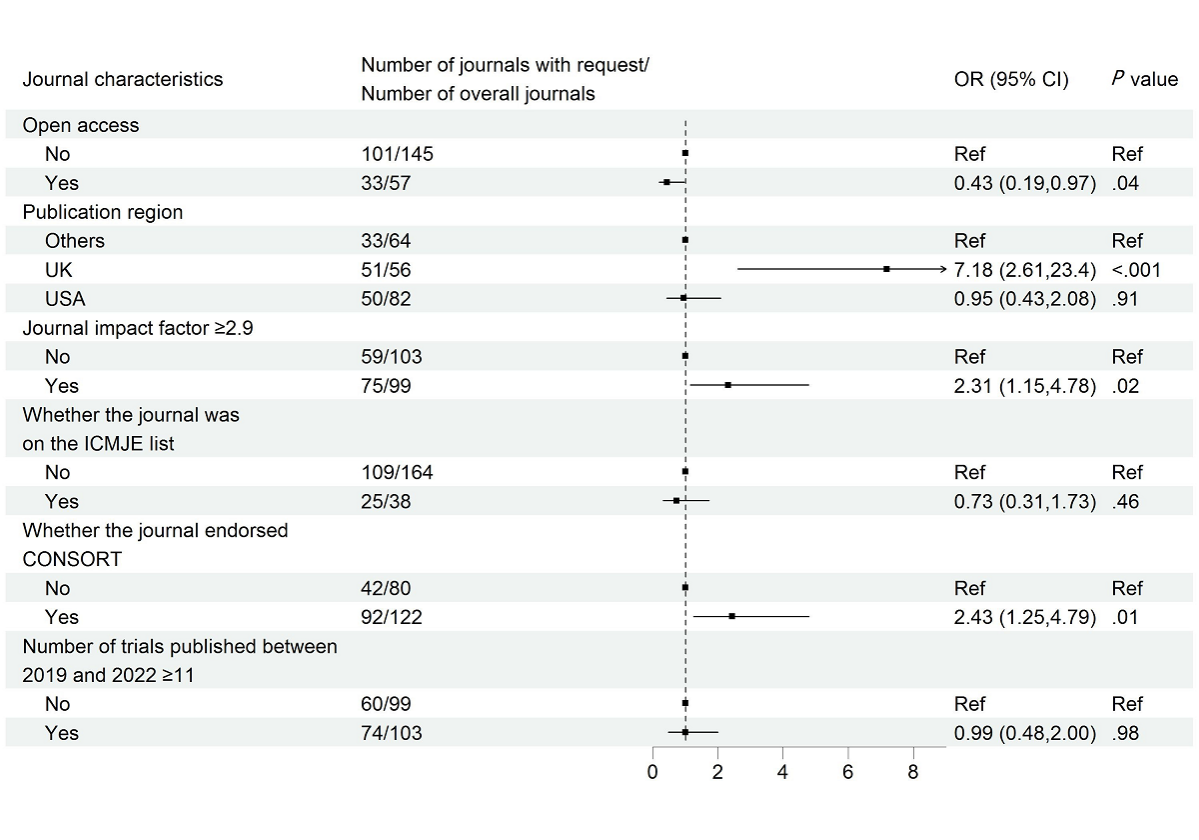
Multivariable logistic regression assessing the association between journal characteristics and request for data-sharing statements. CONSORT: Consolidated Standards of Reporting Trials; ICMJE: International Committee of Medical Journal Editors.

Figure 2 demonstrates results for the relationship between journal characteristics and journal requests for data-sharing statements. Being open access was significantly associated with lower odds of journal requests for data-sharing statements (OR 0.43, 95% CI 0.19-0.97), while journal impact factor ≥2.9 and endorsement of CONSORT were significantly associated with increased odds (OR 2.31, 95% CI 1.15-4.78 and OR 2.43, 95% CI 1.25-4.79, respectively). Compared with other publication regions, UK journals had significantly higher odds of journal request (OR 7.18, 95% CI 2.61-23.40), while no significant relationship was observed in US journals (OR 0.95, 95% CI 0.43-2.08). Results from sensitivity analyses by using the continuous forms of 3 variables (percentage of open access, journal impact factor, and number of trials published between 2019 and 2022) and the GEE approach were largely in line with our main findings (Figures S4 and S5 in Multimedia Appendix 1).

**Figure 3:**
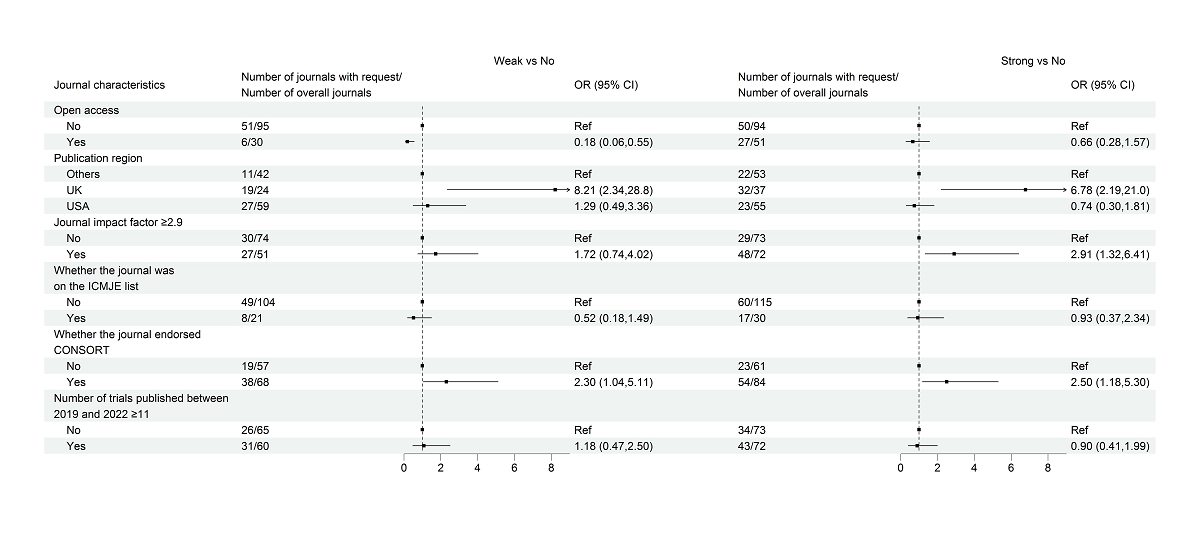
Multinomial logistic regression assessing the association between journal characteristics and two types of journal requests for data-sharing statements. CONSORT: Consolidated Standards of Reporting Trials; ICMJE: International Committee of Medical Journal Editors.

Among the 134 journals requesting data-sharing statements, there were 57 (42.5%) and 77 (57.5%) journals categorized as weak and strong, respectively. Figure 3 shows the results for journal characteristics in relation to the 2 types of journal requests. Both the comparisons between journals with weak requests and those with no requests, and between journals with strong requests and those with no requests, yielded similar results to our main findings.

A significant discordance was observed between journal requests identified on the paper submission instructions and the publication of data-sharing statements identified from published clinical trial reports (*P*<.001, Table 2). Among the 134 journals requesting data-sharing statements, 36 (26.9%) journals did not have statements in the clinical trial reports, while there were 92.6% of the journals without request (63/68) that did not present statements in trial reports. The findings of the association between journal characteristics and journals with any data-sharing statement in their published clinical trial reports were largely consistent with the main results (Figure S6 in Multimedia Appendix 1).

**Table 2:** Data-sharing statements in published clinical trial reports versus journal requests for data-sharing statements identified on the paper submission instructions. Results are shown as count (%) unless otherwise specified.

			Whether the journal requested data-sharing statements on the paper submission instructions				Total
			No	Yes			
**Whether there was any** **data-sharing** **statement in clinical trial reports published in the journal, n (%)**							
	None	63 (31.2)		36 (17.8)	99 (49)		
	Any	5 (2.5)		98 (48.5)	103 (51)		
Total			68 (33.7)	134 (66.3)		202 (100)	

There was a total of 7 journals included in both Siebert’s study and our own (Table S2 in Multimedia Appendix 1). One journal (out of 7 journals, 14.3%), the *International Journal of Epidemiology*, that did not request data-sharing statements during Siebert’s study, was found to have changed to requesting data-sharing statements at the time of our survey. However, the difference in journal requests was not statistically significant (*P*=.50).

## Discussion

### Principal Findings

In this survey, we explored the data-sharing statement request of public, environmental, and occupational health journals. The principal findings were as follows: (1) over one-third of journals did not request data-sharing statements in clinical trial reports; (2) being open access was significantly associated with decreased odds of journal request, while publication in the United Kingdom, higher journal impact factor, and endorsement of CONSORT were significantly associated with increased odds of journal requests; and (3) among the journals requesting data-sharing statements, approximately 27% had published trial reports that indeed had no data-sharing statements.

Despite the ICMJE recommendation, our study showed over one-third of the included journals did not request data-sharing statements on their web pages. This data-sharing statement request remained inadequate for public, environmental, and occupational health journals, even though the importance of sharing IPD in public health and health informatics had been prominently highlighted during the COVID-19 pandemic [25]. Ramdjee et al conducted a study to include 148 COVID-19 interventional phase 3 trials registered between September 1, 2020, and March 1, 2021, reporting that the intent to share IPD at registration did not differ between these COVID-19 trials and 296 contemporary non–COVID-19 trials [12]. Furthermore, their subgroup analysis showed that among vaccine trials, COVID-19 trials were significantly related to decreased odds of intent to share IPD when compared with non–COVID-19 trials (OR 0.08, 95% CI 0.01-0.46). Likewise, another study that included 36 COVID-19 vaccine trial reports found that 86% of trials reported data-sharing statements [26]. Even though all 36 trials were from 3 high-impact journals (*New England Journal of Medicine*, *The Lancet*, and *The Lancet Infectious Diseases*), representing the best-case scenario, this again indicated a suboptimal practice in journal requests for data-sharing statements. Vaccines are known as the most effective strategy to safeguard public health especially during a pandemic, while COVID-19 vaccine trials have received major concerns over their data integrity [27-30]. Therefore, more efforts are urgently needed to enhance journal requests for data-sharing statements and thus the actual IPD sharing in trials in the field of public, environmental, and occupational health.

In our exploratory analysis, we observed that 1 journal that did not request data-sharing statements in 2018 changed to requesting data-sharing statements in 2023 [15]. This may reflect a potential improvement toward journal requests in public, environmental, and occupational health journals temporally. Nonetheless, the small sample size may tend to yield spurious findings by chance that should thus be interpreted with caution.

Our study revealed that among the journals on the ICMJE list, more than one-third did not request data-sharing statements. Furthermore, being on the ICMJE list was associated with decreased odds of journal requests for data-sharing statements, although the relationship was not statistically significant (Figure 2). Despite the ICMJE acknowledging that “there may be some listed journals that do not follow all of the many recommendations and policies in the document,” more actions would be needed to enhance the request and eventually promote the actual IPD sharing among the ICMJE-listing journals [31]. Open access journals were less likely to request data-sharing statements than subscription journals, in line with a previous study [32]. Some open access journals may be reluctant to have data-sharing requests, because this may further increase trial authors’ burden besides costly publication fees [33]. Furthermore, studies suggested that open access journals might lack the resources to enforce data sharing [32,33], which may partly explain the decreased odds of requesting data-sharing statements in open access journals.

Although the United States and the United Kingdom had the greatest numbers of public, environmental, and occupational health journals, our study showed that UK-based journals were more likely to request data-sharing statements. Nevertheless, guidelines on data sharing have been established by the national agencies in both the United Kingdom and the United States [34,35]. The elevated odds of requesting data-sharing statements in UK journals may be due to a combination of regulatory frameworks (ie, UK Research and Innovation Guidelines and General Data Protection Regulation), encouraging attitudes toward open science, and more support from academic institutions. Another possible explanation was that a higher distrust of sharing data had been reported in the United States than in the United Kingdom [4]. Besides, journal endorsement of CONSORT and a higher journal impact factor were associated with increased odds of journal requests for data-sharing statements. Journals with a higher impact factor were found to generally have more stringent peer-review processes and higher-quality publications [36]. Similarly, trial reports published in journals with the requirement of adhering to the CONSORT checklist were typically of higher quality than those trial reports published in journals without the CONSORT requirement [37]. However, these results should be treated with caution, especially given the existence of potential residual confounding effects and unmeasured biases from a nonrandomized survey.

Notably, we found a significant discordance between journal requests on the paper submission instructions and journal practice of including a data-sharing statement in their published trial reports (Table 2). Again, this information emphasized a large room for improving the current practice of journal requests, even in those journals that declared to request trial authors to provide data-sharing statements on the paper submission instructions.

Some previous studies have explored journal requests for data-sharing statements; however, none of these studies focused on public, environmental, and occupational health journals [13-16]. For example, a recent study included top journals in each quartile from 178 categories to examine requests for data-sharing statements in journals of life, health, and physical sciences [16]. While we ran a post hoc analysis by matching their included journals with ours, there were only 5 public, environmental, and occupational health journals included in their study. Similarly, to the best of our knowledge, the survey by Siebert et al involved the largest number of biomedical journals among previous studies, yet it only included 7 public, environmental, and occupational health journals for analysis [15].

Therefore, our survey may provide comprehensive evidence of whether public, environmental, and occupational health journals requested data-sharing statements in clinical trial submissions, thereby potentially generating some new insights into enhancing reporting transparency and eventually improving the actual sharing of IPD in clinical trials. The risk factors identified in this study might be targeted to improve the deficiency of current data-sharing statement request practice. Effective communication between publishers, journals, authors, academic institutions, and funders is encouraged to reach a potential consensus on data-sharing statement requests. Some perspectives on improving data-sharing statement requests may be considered. Publishers and journals may consider applying strict enforcement of their data-sharing statement request and actively monitoring compliance with it. Assigning specific human resources for evaluating data-sharing statements and actual data-sharing could be another possible option. Funders may provide user-friendly data-sharing platforms or tools and specific strategies to encourage data sharing. Editors and peer reviewers need to be tasked with evaluating the adequacy of data-sharing statements provided in submitted trial papers. The societies and associations in the field of public, environmental, and occupational health may consider establishing resource-sharing alliances for scholars and prioritizing publishing incentive policies, aiming to promote a culture of collaboration and openness in human research. Researchers are encouraged to adopt a data management plan at the beginning of a trial and closely adhere to their plans. Overall, joint endeavors from multiple stakeholders would be required to improve the journal request for data-sharing statements and to address the important gap between declared data-sharing statement requests in journals’ webpages and the actual statements provided in their published trial reports, ultimately promoting advancement, information transparency, and reproducibility in public, environmental, and occupational health research.

### Strengths and Limitations

This survey was the first to systematically explore whether public, environmental, and occupational health journals requested data-sharing statements in their clinical trial report submissions. Rigorous methodology and detailed analyses strengthened our findings.

Several limitations need to be noted. First, no causal effects of journal characteristics on journal requests for data-sharing statements could be obtained in this observational study. Similarly, potential biases and confounding could not be fully precluded. As Google Translate was used in our study, potential errors due to misinterpretation of non-English or Chinese-language journals (n=8) could not be addressed. In addition, some journals involving multiple fields of health research (eg, *The New England Journal of Medicine* and *The Lancet*) that were not in the category of “Public, Environmental, and Occupational Health” indeed frequently published trials in the field of public, environmental, and occupational health. Even though we aimed to target public, environmental, and occupational health journals by using the definition of Journal Citation Reports for inclusion, this may fail to include a comprehensive list of public, environmental, and occupational health-related journals for analysis. Moreover, we did not contact journal editors or publishers who may determine or at least get involved in making the request for data-sharing statements to further verify our study outcome, mainly due to the low feasibility. Nevertheless, the results of our mockup submission in a random sample of journals without request identified from their submission guidance pages could partly support the accuracy of our outcome measures. Notably, these findings could only reflect the practice of journal requests for data-sharing statements during the time of our study. Given the dynamic change in policy endorsement, future explorations are needed to investigate the latest practice of journal requests for data-sharing statements.

### Conclusions

Over one-third of the public, environmental, and occupational health journals did not request data-sharing statements in clinical trial reports. Among those journals that requested data-sharing statements in their submission guidance pages, more than one quarter published trial reports with no data-sharing statements. These results revealed an inadequate practice of requesting data-sharing statements by public, environmental, and occupational health journals, requiring more efforts at the journal level to implement ICJME recommendations on data-sharing statements.

## Appendix

Multimedia Appendix 1 Supplemental materials.

## Abbreviations

CONSORT: Consolidated Standards of Reporting Trials
GEE: generalized estimating equations
ICMJE: International Committee of Medical Journal Editors
IPD: individual patient data
OR: odds ratio
STROBE: Strengthening the Reporting of Observational Studies in Epidemiology
VIF: variance inflation factor

## Data Availability

The datasets generated during and/or analyzed during this study are available from the corresponding author on reasonable request.
