# Supplementary material for: Data-Sharing Statements Requested from Clinical Trials by Public, Environmental, and Occupational Health Journals: Cross-Sectional Study
Source: J Med Internet Res. 2025 Feb 7;27:e64069. doi: 10.2196/64069 (PMC11845885; doi:10.2196/64069)
Supplement: Multimedia Appendix 1 [file jmir_v27i1e64069_app1.docx]

**Supplemental materials**

[**SFigure 1**. Flow diagram showing journal selection process for this study 2](#_Toc169884525)

[**SFigure 2**. Ranking of the number of included journals from corresponding publication regions 3](#_Toc169884526)

[**SFigure 3**. Ranking of the number of included journals from corresponding publishers 4](#_Toc169884527)

[**SFigure 4.** Sensitivity analysis results for the association between journal characteristics and request for data sharing statements when treating Open Access, Journal Impact Factor, number of trials as continuous variables 5](#_Toc169884528)

[**SFigure 5.** Results for the association between journal characteristics and request for data sharing statements using the GEE approach 6](#_Toc169884529)

[**SFigure 6.** Results for the association between journal characteristics and journals with Any data sharing statement in their published clinical trial reports 7](#_Toc169884530)

[**STable 1**. List of descriptions of data sharing statement request identified on the manuscript submission instructions 8](#_Toc169884531)

[**STable 2.** Exploratory analysis results from comparing the previous study published in BMJ Open with our current study regarding the number of journals according to data sharing statement request* 10](#_Toc169884532)

Journals in the category of “Public, Environmental & Occupational Health” and in the edition of “SCIE” defined by the Journal Citation Reports (n=207)

Journals after duplicates removed (n = 400)

Journals included for analysis
(n = 202)

Journals were excluded because they did not publish clinical trial reports with IPD between 2019 and 2022 after searching the journal webpages (n = 198)

Journals in the category of “Public, Environmental & Occupational Health” and in the edition of “SSCI” defined by the Journal Citation Reports (n=181)

Journals in the category of “Public, Environmental & Occupational Health” and in the edition of “ESCI” defined by the Journal Citation Reports (n=100)

**SFigure 1**. Flow diagram showing journal selection process for this study


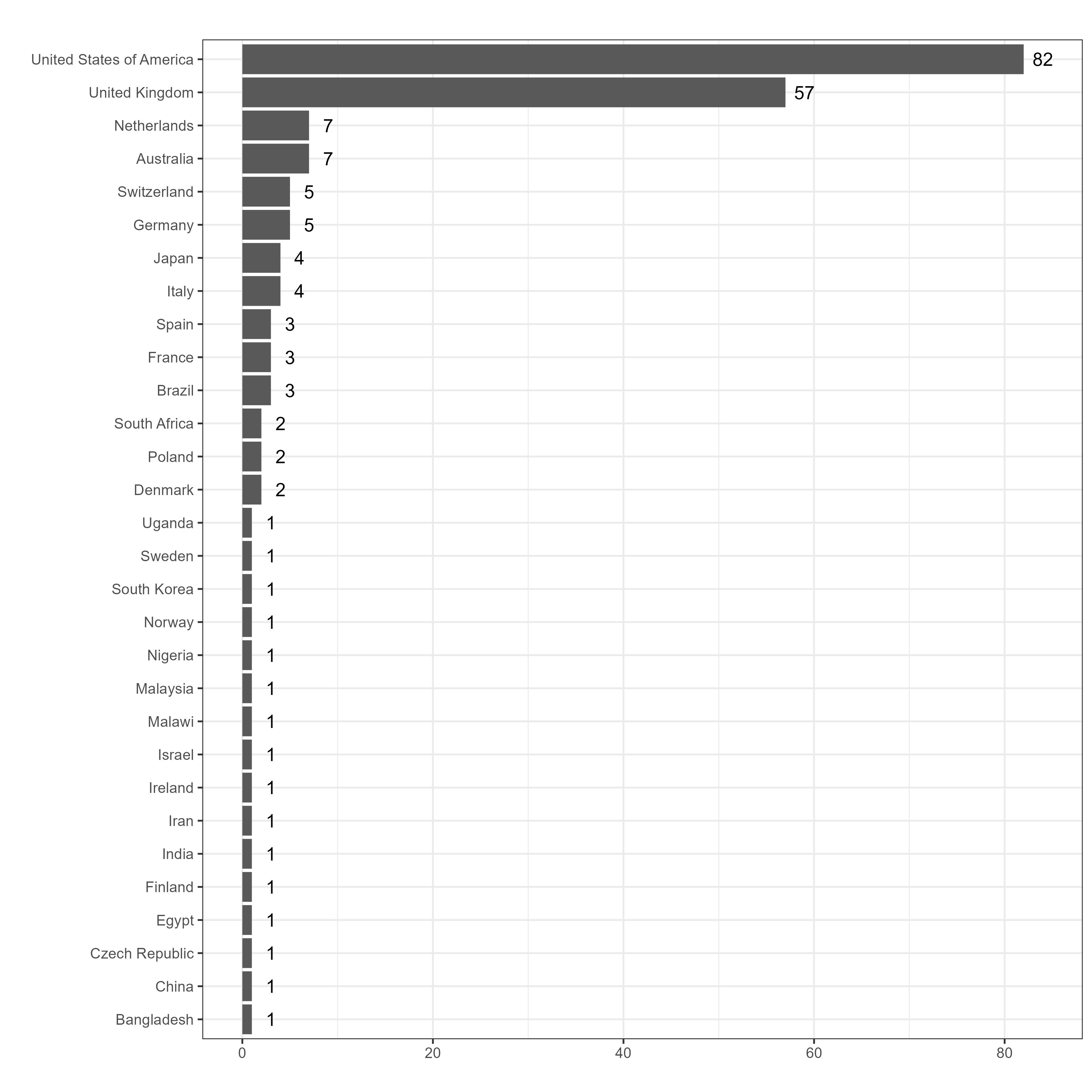


**SFigure 2**. Ranking of the number of included journals from corresponding publication regions


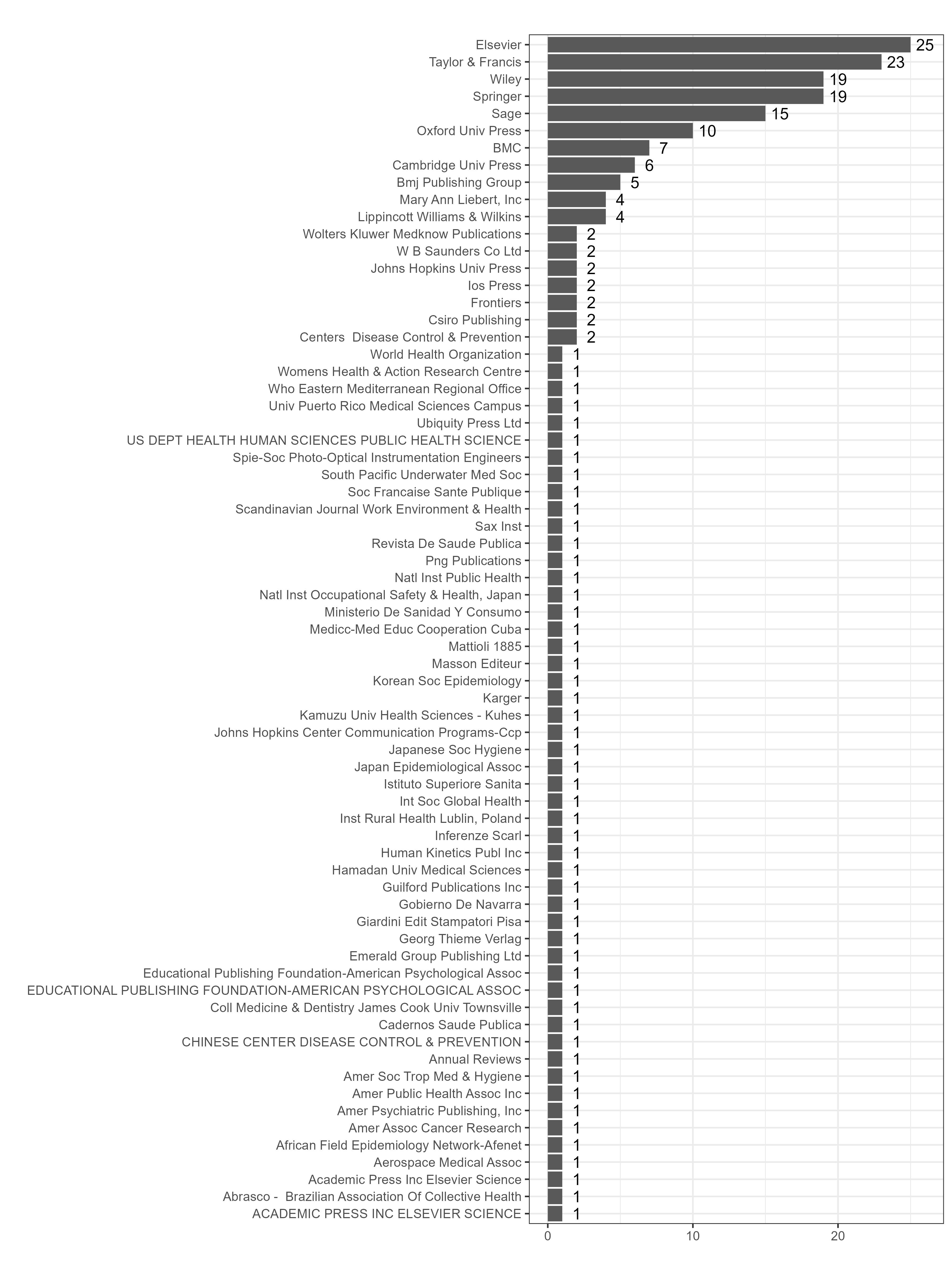


**SFigure 3**. Ranking of the number of included journals from corresponding publishers


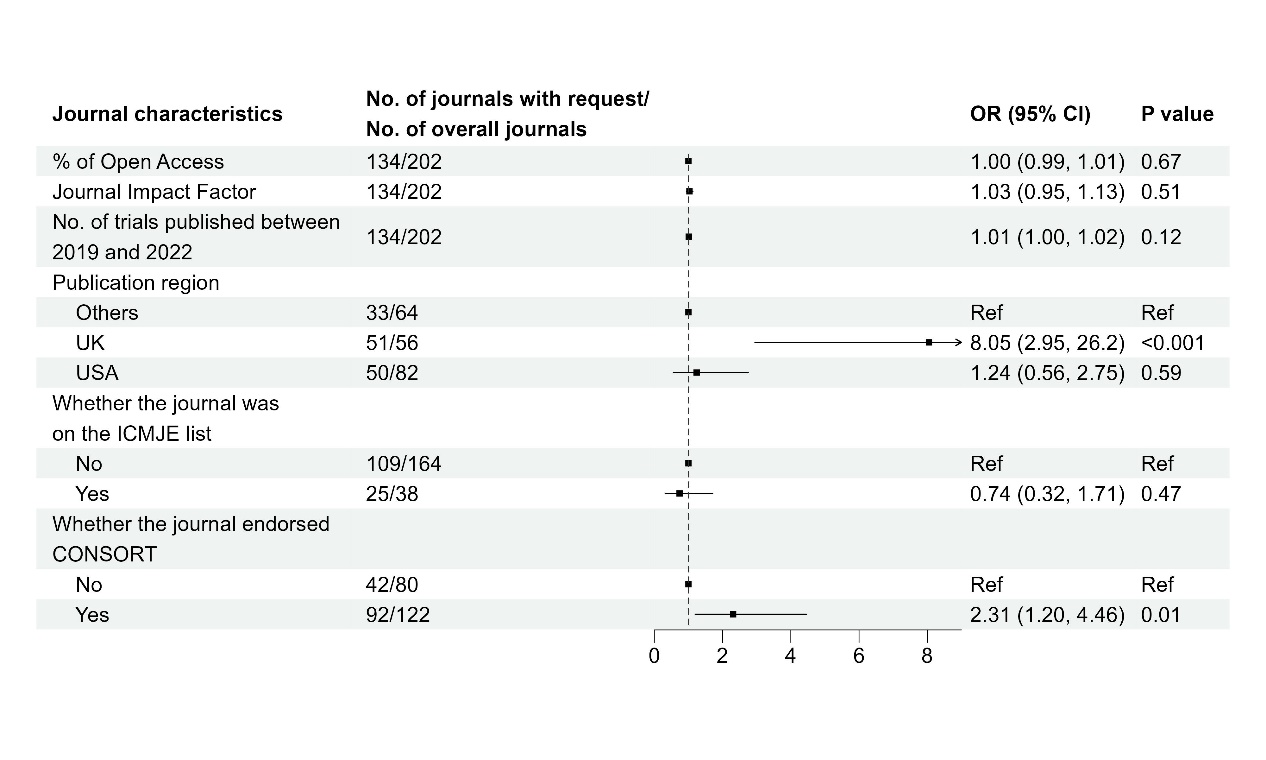


**SFigure 4.** Sensitivity analysis results for the association between journal characteristics and request for data sharing statements when treating Open Access, Journal Impact Factor, number of trials as continuous variables

CONSORT (Consolidated Standards of Reporting Trials); ICMJE (International Committee of Medical Journal Editors); OR (Odds Ratio); CI: Confidence Interval


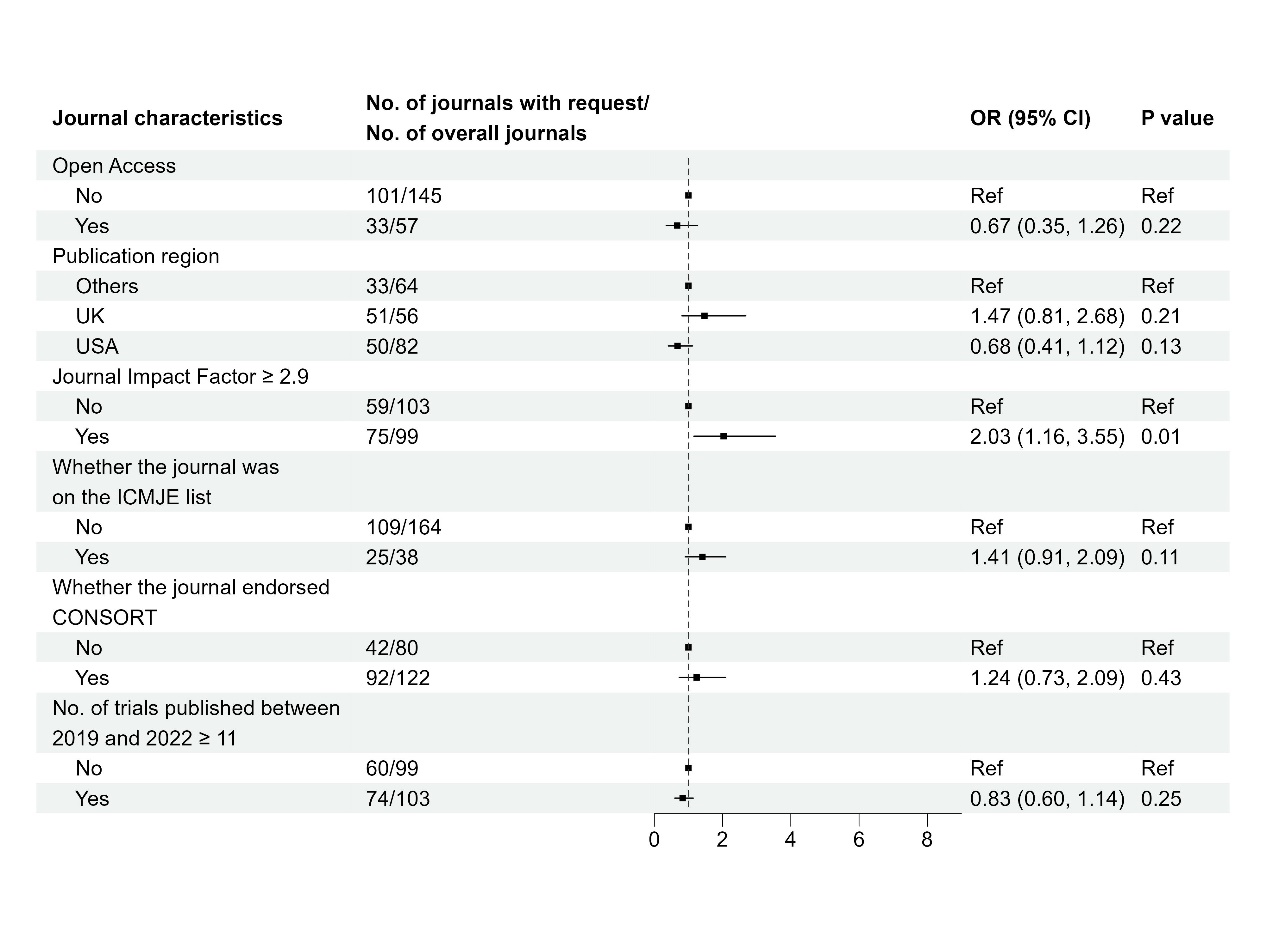


**SFigure 5.** Results for the association between journal characteristics and request for data sharing statements using the GEE approach

Note: The median Journal Impact Factor was 2.9; the median number of trials among all the journals was 11.0.

CONSORT (Consolidated Standards of Reporting Trials); ICMJE (International Committee of Medical Journal Editors); OR (Odds Ratio); CI: Confidence Interval; GEE: Generalized Estimating Equations


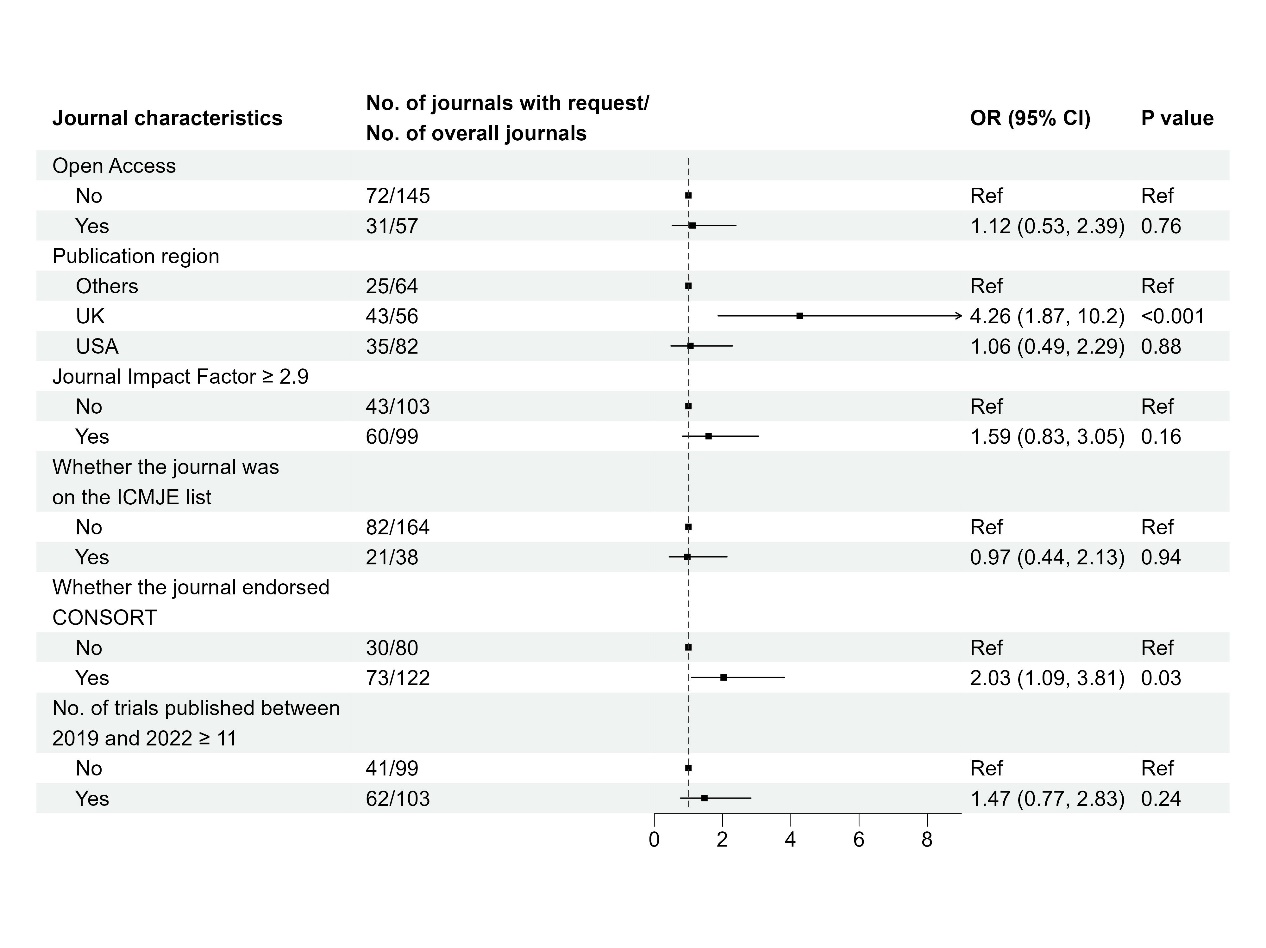


**SFigure 6.** Results for the association between journal characteristics and journals with Any data sharing statement in their published clinical trial reports

Note: The median Journal Impact Factor was 2.9; the median number of trials among all the journals was 11.0.

CONSORT (Consolidated Standards of Reporting Trials); ICMJE (International Committee of Medical Journal Editors); OR (Odds Ratio); CI: Confidence Interval

**STable 1**. List of descriptions of data sharing statement request identified on the manuscript submission instructions

| **Type** | **Term** | **Keywords** | **Descriptions** |
| --- | --- | --- | --- |
| Weak | Encourage | Data accessibility | The journal encourages authors to share their research data in a suitable public repository subject to ethical considerations and where data is included, to add a **data accessibility statement** in their manuscript file. |
|  |  | Data availability | Authors are further encouraged to cite any data sets referenced in the article and provide a **Data Availability Statement**. |
|  |  |  | **Data availability:** This statement should describe how readers can access the data supporting the conclusions of the study and clearly outline the reasons why unavailable data cannot be released. |
|  |  |  | The journal encourages authors to provide a **statement of Data availability**. |
|  |  |  | To foster transparency, we encourage you to state the **availability** of your data in your submission. |
|  |  | Data availability/sharing | For original research, authors are encouraged to provide **data availability** or **data sharing** statement. |
|  |  | Data deposit | The journal encourages authors, where possible and applicable, to **deposit data** that support the findings of their research in a public repository. |
|  | Recommend | Data availability | We recommend that a **data availability statement** be included in the manuscript in the Methods section or as a separate section at the end of the main text file. |
|  |  | Data sharing | The journal recommends that all submitted manuscripts that report the results of clinical trials adhere to **Data Sharing Statements** for Clinical Trials: A Requirement of the International Committee of Medical Journal Editors. |
| Strong | Mandate | Data deposition | The journal mandates **data deposition.** |
|  | Must | Data availability | A **Data Availability Statement** must be included as part of your manuscript. |
|  |  |  | All original research must include a **data availability statement**. |
|  |  |  | Sharing of all relevant research data is strongly encouraged and authors must add a **Data Availability Statement** to original research articles. |
|  |  | Data sharing | All manuscripts reporting the results of clinical trials must include a **data sharing** plan in the Acknowledgments section of the manuscript. |
|  |  |  | All submitted research Articles must contain a **data sharing statement**, to be included at the end of the manuscript. |
|  |  |  | As per the ICMJE recommendation, adopted by the journal, clinical trials must contain a **data sharing statement**. |
|  |  |  | Manuscripts reporting on clinical trial data must contain a **data sharing statement.** |
|  | Request | Data availability | During submission, you will be requested to reply to a series of questions on the **availability of the data** used for the research. Based on your answers, a **data availability statement** will be generated and added to your manuscript. |
|  | Require | Data accessibility | The journal requires authors of original research studies to describe in their papers how readers can **access** the data. |
|  |  | Data availability | A **Data Availability Statement** is required by the journal. |
|  |  |  | Authors are required to provide a **Data Availability Statement.** |
|  |  |  | **Data availability** declarations are required under research data policy types. |
|  |  |  | Please include a **statement of data availability.** |
|  |  |  | The inclusion of a **data availability statement** is a requirement for papers published in the Journal. |
|  |  |  | The journal requires that authors provide a statement on the **availability of data**. |
|  |  |  | To foster transparency, we require you to state the **availability of your data** in your submission. |
|  |  | Data sharing | Clinical research studies require a **data sharing statement.** |
|  |  |  | The journal now requires authors to explain their **data sharing plan** by including weblinks (preferably DOIs) to source data and data sets, following the SUPPLEMENTAL MATERIAL section. |
|  |  |  | This journal expects **data sharing.** |
|  | Should | Data accessibility | Authors should include a **data accessibility statement.** |
|  |  | Data availability | Authors should include a **Data Availability Statement.** |
|  |  |  | **Data availability statements** for manuscripts reporting clinical trial data should follow the standards set out in the ICMJE recommendations on clinical trial data sharing. |
|  |  |  | **Data availability statements** should be placed under the heading “Data and Resource Availability” at the end of the “Research Design and Methods” section. |
|  |  |  | For all manuscripts, information about **data availability** should be detailed in an ‘Availability of data and materials’ section. |

**STable 2.** Exploratory analysis results from comparing the previous study published in BMJ Open with our current study regarding the number of journals according to data sharing statement request*

|  | | **Journals according to data sharing statement request in the BMJ Open study** | | **Total** |
| --- | --- | --- | --- | --- |
|  |  | No request | With request |  |
| **Journals according to data sharing statement request in our current study** | No request | 1 (14.3) | 0 (0) | 1 (14.3) |
|  | With request | 1 (14.3) | 5 (71.4) | 6 (85.7) |
| **Total** | | 2 (28.6) | 5 (71.4) | 7 (100.0) |

* Results shown as count (%) unless otherwise specified

[1] Siebert M, Gaba JF, Caquelin L, et al. Data-sharing recommendations in biomedical journals and randomized controlled trials: an audit of journals following the ICMJE recommendations. BMJ Open 2020;10:e038887. doi:10.1136/bmjopen-2020-038887
